# Clinical, Endoscopic, and Histological Characteristics of Severe Immune Checkpoint Inhibitor-Induced Colitis

**DOI:** 10.3390/jcm15010353

**Published:** 2026-01-02

**Authors:** Diego Casas Deza, Cristina Polo Cuadro, Marta Gascón Ruiz, Manuel Barreiro-de Acosta, Míriam Mañosa, Francisco Rodríguez-Moranta, Yamile Zabana, Elena Céspedes Martínez, Ingrid Ordás, José Miranda Bautista, María José García, Irene García de la Filia Molina, Cristina Roig Ramos, Alexandra Ruiz Cerulla, José Xavier Segarra-Ortega, Virginia Matallana Royo, Esther Rodríguez González, Fernando Martínez de Juan, Noemí Manceñido Marcos, Lucía Madero Velázquez, Elena Betoré Glaría, Begoña Álvarez Herrero, Gerard Suris, Alejandro Garrido Marín, Eduard Brunet Mas, Inmaculada Alonso Abreu, Javier Santos Fernández, María Vaamonde Lorenzo, Cristina Almingol Crespo, Carla Folguera, Patricia Sanz Segura, Óscar Moralejo Lozano, Laura López Couceiro, Coral Tejido Sandoval, Raquel Mena Sánchez, Empar Sainz, Miquel Marquès-Camí, Rocío Ferreiro-Iglesias, Silvia Patricia Ortega Moya, Pablo Miles Wolfe García, Pere Borras Garriga, Belén Herreros Martínez, María Calvo Iñiguez, Santiago Frago Larramona, Pablo Ladrón Abia, Xavier Serra-Ruiz, Luis Menchén, Coral Rivas Rivas, Francisco Mesonero Gismero, Raquel Vicente Lidón, Ana Gutierrez, Santiago García López

**Affiliations:** 1 Servicio de Aparato Digestivo, Hospital Universitario Miguel Servet, Instituto de Investigación Sanitaria de Aragón, 50009 Zaragoza, Spain; 2 Servicio de Oncología Médica, Hospital Universitario Miguel Servet, Instituto de Investigación Sanitaria de Aragón, 50009 Zaragoza, Spain; marta.gascon6@gmail.com; 3 Servicio de Aparato Digestivo, Hospital Clínico Universitario de Santiago de Compostela, Instituto de Investigación Sanitaria de Santiago de Compostela (IDIS), 15706 Santiago de Compostela, Spain; 4 Servicio de Aparato Digestivo, Hospital Universitari Germans Trias i Pujol, 08916 Badalona, Spain; 5 Servicio de Aparato Digestivo, Hospital Universitari de Bellvitge, 08907 L’Hospitalet de Llobregat, Spain; frmoranta@bellvitgehospital.cat; 6 CIBERhed, Hospital Universitari Mútua de Terrassa, 08221 Terrassa, Spain; 7 Unitat d’Atenció Crohn-Colitis (UACC), Servicio de Aparato Digestivo, Hospital Universitario Vall d’Hebron, 08035 Barcelona, Spainxavier.serra@vallhebron.cat (X.S.-R.); 8 Unidad de Enfermedad Inflamatoria Intestinal, Servicio de Gastroenterología, Hospital Clínic, IDIBAPS, CIBERehd, 08036 Barcelona, Spain; 9 Servicio de Aparato Digestivo, Hospital General Universitario e Instituto de Investigación Sanitaria Gregorio Marañón, 28009 Madrid, Spain; pepon_miranda@hotmail.com (J.M.B.);; 10 Hospital Universitario Marqués de Valdecilla, IDIVAL, 39008 Santander, Spain; 11 Hospital Universitario Ramón y Cajal, 28034 Madrid, Spain; 12 Servicio de Patología digestiva, Hospital Universitari de la Santa Creu i Sant Pau, 08025 Barcelona, Spain; 13 Hospital Universitario de Bellvitge, 08097 Hospitalet de Llobregat, Spain; 14 Hospital Universitario de Salamanca, 37007 Salamanca, Spain; mdjosexaviersegarra9@gmail.com; 15 Servicio de Aparato Digestivo, Hospital Universitario Puerta de Hierro, 28222 Majadahonda, Spain; 16 Hospital Nuestra Señora de la Candelaria, 38010 Tenerife, Spain; gerodgon@gmail.com; 17 Unidad de Aparato Digestivo, Instituto Valenciano de Oncología, 46009 Valencia, Spain; 18 Servicio de Servicio de Aparato Digestivo, Hospital Universitario Infanta Sofía, Facultad de Medicina, Salud y Deportes, Departamento de Medicina, Universidad Europea de Madrid, FIIB HUIS-HUHEN, 28709 San Sebastián de los Reyes, Spain; 19 Hospital General Universitario Dr. Balmis, ISABIAL, 03010 Alicante, Spain; 20 Hospital Universitario San Jorge, 22004 Huesca, Spain; 21 Servicio de Aparato Digestivo, Hospital Universitario de Álava, 01009 Vitoria-Gasteiz, Spain; 22 Unidad de Enfermedad Inflamatoria Intestinal, Servicio de Aparato Digestivo, Hospital del Mar, 08003 Barcelona, Spain; 23 Hospital Universitario La Fe, 46026 Valencia, Spain; 24 Servicio de Aparato Digestivo, Hospital Universitari Parc Taulí, CIBERehd, 08208 Sabadell, Spain; 25 Unidad de EII, Servicio de Aparato Digestivo, Hospital Universitario de Canarias, 38320 Tenerife, Spain; 26 Servicio de Aparato Digestivo, Complejo Asistencial Universitario de Palencia, 34005 Palencia, Spain; 27 Servicio de Aparato Digestivo, Hospital Universitario de Donostia, 20014 San Sebastián, Spain; 28 Hospital Universitario Galdakao-Usansolo, 48960 Galdakao, Spain; 29 Servicio de Aparato Digestivo, Hospital Joan XXIII, 43007 Tarragona, Spain; 30 Servicio de Aparato Digestivo, Hospital Royo Villanova, 50015 Zaragoza, Spain; 31 Servicio de Aparato Digestivo, Hospital Universitario de Toledo, 45007 Toledo, Spain; oscarmoralejo@gmail.com; 32 Servicio de Aparato Digestivo, Hospital Universitario Infanta Cristina, 28981 Parla, Spain; 33 Complejo Hospitalario Universitario de Ourense, 32005 Ourense, Spain; 34 Unidad de Enfermedad Inflamatoria Intestinal, Hospital Universitari Consorci Sanitari de Terrassa, 08222 Barcelona, Spain; 35 Xarxa Assistencial Universitaria Althaia, 08243 Manresa, Spain; 36 Hospital Universitari Arnau de Vilanova, 25198 Lleida, Spain; 37 Hospital Comarcal de Inca, 07300 Inca, Spain; 38 Servicio de Aparato Digestivo, Hospital de Sierrallana, 39300 Torrelavega, Spain; 39 Servicio de Aparato Digestivo, Hospital Universitari Mútua de Terrassa, 08221 Terrassa, Spain; 40 Hospital Marina Baixa, 03570 Villajoyosa, Spain; 41 Servicio de Aparato Digestivo, Hospital de Logroño, 26006 Logroño, Spain; 42 Servicio de Aparato Digestivo, Complejo Asistencial de Soria, 42005 Soria, Spain; 43 Servicio de Aparato Digestivo, Hospital Universitario de Burgos, 09006 Burgos, Spain; 44 Hospital General Universitario Dr. Balmis, ISABIAL, CIBERehd, 03010 Alicante, Spain

**Keywords:** immune checkpoint inhibitors, immune-mediated colitis, endoscopy, histology

## Abstract

**Background:** Immune checkpoint inhibitors (ICIs) have revolutionized cancer therapy. They can cause immune-mediated colitis (IMC), a potentially severe adverse event. Current data on severe IMC (grade 3–4) are limited, particularly regarding clinical, endoscopic, and histological features. **Methods:** We conducted a multicenter, retrospective observational study promoted by GETECCU, including adults with solid tumors who developed grade 3–4 IMC requiring hospitalization and systemic therapy. Clinical symptoms, endoscopic findings (Mayo and UCEIS indices), and histological features were systematically collected and analyzed. **Results:** A total of 196 patients were included. Diarrhea was universal (median 8 bowel movements/day), with 76% reporting abdominal pain and 39% rectal bleeding. Endoscopy (n = 139) revealed vascular pattern loss (80%), mucosal lesions (69%), and Mayo scores ≥2 in 69%. Histopathology (n = 141) showed abnormalities in 85%, including cryptitis (50%), lymphocytic infiltration (48%), and crypt abscesses (37%). Notably, 72% of patients with normal endoscopy had histological inflammation. Endoscopic severity correlated with bleeding and impaired general condition but not with stool frequency or pain intensity. Histological and endoscopic severity were modestly associated. **Conclusions:** Severe IMC presents with heterogeneous clinical, endoscopic, and histological features, with limited correlation between these domains. Endoscopic findings were often ulcerative and inflammatory, yet histological abnormalities were frequently present even in endoscopically inactive disease. These findings highlight the importance of biopsy in all suspected IMC cases and underscore the need for validated multidimensional assessment tools for accurate diagnosis and management of severe IMC.

## 1. Introduction

Immune checkpoint inhibitors (ICIs) have transformed cancer therapy by enhancing antitumor immune responses, including agents targeting cytotoxic T-lymphocyte-associated antigen 4 (CTLA-4), programmed cell death protein 1 (PD-1), and its ligand (PD-L1). However, this mechanism also predisposes to immune-related adverse events (irAEs) affecting multiple organs, among which gastrointestinal toxicity is particularly relevant due to its frequency and potential to compromise oncologic outcomes [1,2,3,4,5,6].

Immune-mediated colitis (IMC) is one of the most common and clinically significant irAEs. Its reported incidence ranges from 3% to 30%, depending on the ICI regimen—being higher with anti-CTLA-4 agents and combination therapies [7]. Despite its impact, IMC remains an incompletely characterized entity. The absence of standardized diagnostic criteria complicates early recognition and optimal management. While some endoscopic and histological features have been described [8,9,10,11,12,13,14,15], most studies are retrospective, single-center, and include limited patient numbers. Furthermore, the clinical presentation and severity spectrum have been less consistently reported [7,12,16].

In response to this need, we designed a multicenter, nationwide study promoted by the Spanish Working Group on Crohn’s Disease and Ulcerative Colitis (GETECCU), aimed at systematically characterizing the clinical, endoscopic, and histological findings in patients with moderate to severe flares of immune-mediated colitis (grades 3–4 ac-cording to the Common Terminology Criteria for Adverse Events [CTCAE] v5.0).

## 2. Materials and Methods

### 2.1. Study Design

This was a multicenter, observational, retrospective study promoted by the Spanish Working Group on Crohn’s Disease and Ulcerative Colitis (GETECCU). We included patients aged ≥18 years with a diagnosis of solid organ malignancy treated with immune checkpoint inhibitors in the context of routine clinical practice, who developed grade 3 or 4 immune-mediated colitis according to CTCAE v5.0 criteria from 2014 to 2024. Patients from 39 different centres were included. All patients required hospital admission and received at least one dose of intravenous corticosteroids and/or specific immunosuppressive therapy for IMC. Patients treated within clinical trials, managed exclusively in the outpatient setting, or who did not receive immunosuppressive therapy were excluded. Patients were identified from four main sources: discharge diagnosis codes, hospital pharmacy records, endoscopy unit databases, and pathology service databases.

Almost all participating centres have accredited units for treating patients with inflammatory bowel disease, ensuring quality care. This includes training for gastroenterologists, pathologists, endoscopists, etc., and ensures that the professionals involved in patient management are experienced and well trained.

The diagnosis of IMC was established by the attending oncologist or gastroenterologist based on current clinical criteria and after exclusion of infectious causes (a mandatory inclusion criterion for the registry), including stool cultures and *Clostridioides* difficile testing (GDH negative or GDH positive with PCR and toxin negatives) previously to immunosuppression. Collected variables included demographic data, medical history, oncological characteristics, clinical and laboratory findings at admission, as well as endoscopic and histological findings.

### 2.2. Symptom Assessment

The following symptoms were recorded: number of bowel movements in 24 h, presence of rectal bleeding, abdominal pain, and general condition. Abdominal pain was classified as absent, mild, moderate, or severe; general condition as normal, mildly, moderately, or severely affected. Additionally, the presence of tachycardia, hemodynamic instability, and anemia at admission was documented, along with the time from initiation of immunotherapy to onset of symptoms and the number of treatment cycles received.

To standardize clinical assessment, a “modified Mayo score” was created, based on the partial Mayo score [17] with an added 0–3 point scale to grade abdominal pain intensity (0: absent; 1: mild; 2: moderate; 3: severe). This assessment has not been validated, but it has been included because the CTCAE classification attaches great importance to the presence of abdominal pain. Similarly, the partial Mayo index has not been validated in this scenario, nor has any other tool or score.

### 2.3. Endoscopic Evaluation

Only endoscopic studies performed before starting treatment were included. Endoscopic findings were documented using the criteria of the Mayo Endoscopic Score [18] and the Ulcerative Colitis Endoscopic Index of Severity (UCEIS) [19]. The following features were assessed: loss of vascular pattern (normal, partial, or complete), mucosal bleeding (absent, contact bleeding, mild spontaneous, or significant spontaneous), and mucosal lesions (absent, aphthae, superficial ulcers, deep ulcers).

### 2.4. Histological Evaluation

Only histological studies performed before starting treatment were included. The histological samples were read and analyzed locally. The presence or absence of the following characteristics was categorically evaluated, due to the absence of standardized protocols or classifications: crypt abscesses, cryptitis, lymphocytic infiltration, apoptosis, lamina propria expansion, collagen band thickening, eosinophilic infiltration, and basal plasmacytosis.

### 2.5. Definitions

Colitis severity was defined according to CTCAE v5.0 criteria.

Hemodynamic instability was defined as the presence of at least two of the following: systolic blood pressure < 90 mmHg, heart rate > 100 bpm, or clinical signs of hypoperfusion (altered mental status, mottled skin, etc.).

Anemia was defined as hemoglobin < 12 g/dL in women or <13 g/dL in men.

### 2.6. Statistical Analysis

The data are presented as either mean and standard deviation or median and inter-quartile range for continuous variables based on their distribution, and as the number of cases and percentage for categorical variables; 95% confidence intervals were calculated. The Mann–Whitney U test or the chi square test with Yates correction were used to assess associations between continuous and categorical variables, respectively. The correlation between quantitative variables was assessed using Spearman’s rho. All statistical tests were two-sided. *p* values < 0.05 were considered statistically significant. No methods of correction for multiple comparisons have been used. The analysis was carried out using Jamovi v 2.3.16 software [www.jamovi.org] accessed on 24 February 2025. Missing values were handled using a negative imputation approach whenever feasible, assigning absence or a negative status to missing categorical variables when this could be reliably inferred from the clinical context or data structure. For all other variables where imputation was not appropriate, analyses were conducted using available data only (complete case analysis). This strategy ensured maximal use of the dataset while minimizing potential bias derived from arbitrary imputation.

Study data were collected and managed using REDCap [20] electronic data capture tools hosted at AEG [Asociación Española de Gastroenterología]. REDCap [Research Electronic Data Capture] is a secure, web-based software platform designed to support data capture for research studies, providing: [a] an intuitive interface for validated data capture; [b] audit trails for tracking data manipulation and export procedures; [c] automated export procedures for seamless data downloads to common statistical packages; and [d] procedures for data integration and interoperability with external sources.

## 3. Results

A total of 196 patients were included. High-quality endoscopic data were available for 139 cases, and histological samples were available for 141. As shown in Table 1, 40.3% of patients were women, with a mean age of 62.7 (±11.4) years. The most frequent tumor origins were lung cancer (33.7%) and melanoma (29.6%). Most patients were receiving immunotherapy in a metastatic setting (68.9%). The most common mechanism of action was PD-1 blockade (61.7%), followed by CTLA-4 inhibition (21.4%). Prior use of systemic oral corticosteroids before hospital admission was recorded in 41.3% of patients.

The median time from the start of immunotherapy to symptom onset was 76.5 days (IQR 28–197). The median number of treatment cycles was 4 (IQR 2–8). Time to onset varied according to the type of immunotherapy: IMC occurred earlier in patients treated with anti-CTLA-4 agents compared to other regimens (48 vs. 96 days, *p* = 0.024).

### 3.1. Symptoms

The primary presenting symptom was diarrhea, with a median of 8 (IQR 6–10) bowel movements per day. Abdominal pain was present in 76% of patients and was moderate to severe in 40% of cases. Rectal bleeding was documented in 39% of the cohort.

More than one-quarter (26%) of patients presented with tachycardia on admission, and 8.2% had hemodynamic instability. Based on clinical assessment, 68.2% were classified as having a moderately or severely impaired general condition. These findings are summarized in Table 2.

### 3.2. Endoscopic Findings

Endoscopic evaluation was performed prior to the initiation of intravenous corticosteroids and/or immunosuppressive therapy in 139 patients (84 were full colonoscopies and 55 were sigmoidoscopies). No differences were detected in symptom intensity, patient age, the mechanism of action of the ICI used, or the proportion of haemodynamic instability between patients who underwent endoscopic examination and those who did not. The most common finding was loss of vascular pattern, observed in 80% of cases. Additionally, 53% presented with mucosal bleeding and 69% with mucosal lesions, distributed as superficial ulcers (22%) and deep ulcers (15%). These results can be found in Table 3.

When aligned with endoscopic indices used in ulcerative colitis, 37% of patients had a Mayo endoscopic score of 3, and 32% a score of 2. According to the UCEIS, 22% of cases were classified as moderate and 8% as severe.

### 3.3. Histological Findings

Structured histological reports were available for 141 patients. Histopathological abnormalities were present in 85% of cases. The most frequent findings were cryptitis (50%), lymphocytic infiltration (48%), and crypt abscesses (37%). Apoptosis was noted in 26% of samples, while basal plasmacytosis (6.4%) and eosinophilic infiltration (3.5%) were less common. Data are shown in Table 4.

### 3.4. Endoscopic and Histological Findings by Immunotherapy Mechanism

When comparing endoscopic severity and histological features between patients treated with PD-1/PD-L1 inhibitors and those receiving CTLA-4 inhibitors (alone or in combination), no significant differences were observed, except for a higher frequency of lamina propria expansion in the CTLA-4 group (40% vs. 21%, *p* = 0.03), and a higher prevalence of crypt abscesses in PD1-PDL1 group (23.7% vs. 41.7%, *p* = 0.049).

### 3.5. Symptom–Endoscopy Correlation

First, the association between the severity of endoscopic involvement and the patient’s clinical symptoms was evaluated. Patients with more severe endoscopic findings more frequently presented with rectal bleeding (64.3% vs. 38.8%, *p* = 0.006). Similarly, those with severe endoscopic inflammation more often exhibited a severely impaired general condition (16.7% vs. 1%, *p* = 0.002). Notably, 87.5% of patients in poor general condition had moderate to severe endoscopic severity scores.

In contrast, no significant associations were found between endoscopic severity and either the number of bowel movements or the intensity of abdominal pain.

**Figure 1:**
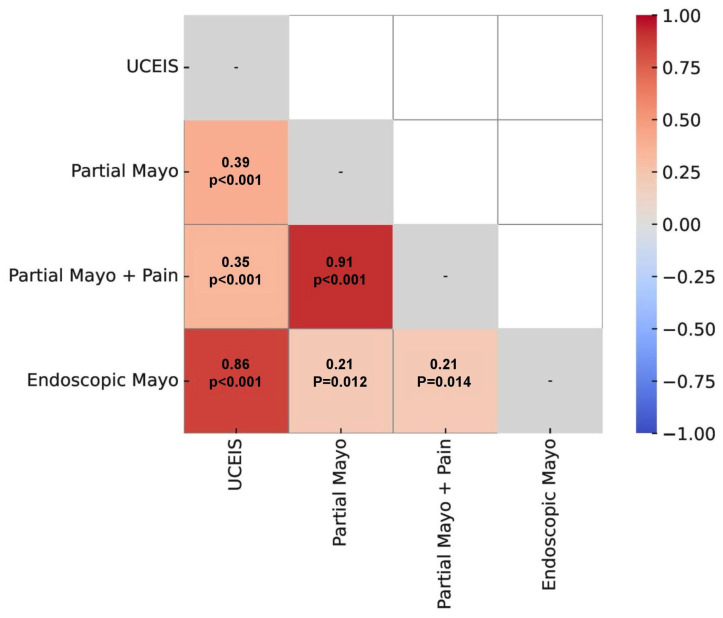
Correlation (Spearman) matrix between clinical and endoscopic indices.

Correlation analysis between clinical indices (partial Mayo score and modified Mayo score including abdominal pain) and endoscopic indices (UCEIS and Mayo endoscopic score) revealed a low clinical–endoscopic correlation, slightly higher for UCEIS. However, both endoscopic indices showed a strong correlation with each other. These results are shown in Figure 1.

### 3.6. Relationship Between Endoscopic and Histological Findings

Paired endoscopic and histological data were available for 133 patients. Cryptitis was more frequent in patients with moderate to severe endoscopic involvement (64.3% vs. 44%, *p* = 0.029). Although all cases of collagen band thickening and eosinophilic infiltration occurred in patients with mild or no endoscopic findings, these differences were not statistically significant (*p* > 0.1). Notably, 72% of patients without macroscopic findings on endoscopy exhibited microscopic abnormalities on histological examination.

**Figure 2:**
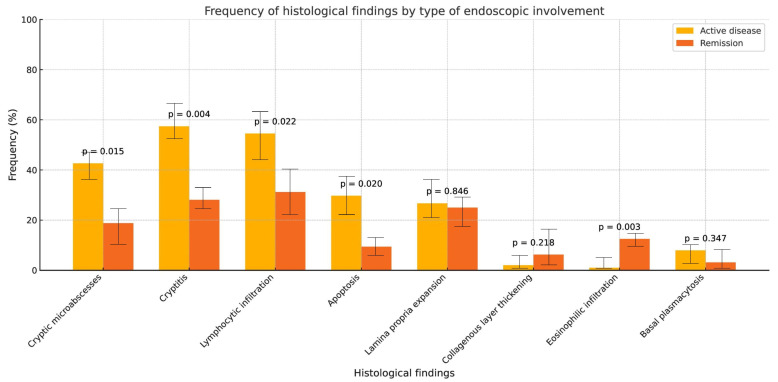
Comparison of histological findings in patients with and without endoscopic activity.

Additionally, when grouping patients with and without endoscopic activity based on the UCEIS score, significant differences were observed. Histological features such as crypt abscesses (42% vs. 19%, *p* = 0.015), cryptitis (57% vs. 28%, *p* = 0.004), lymphocytic infiltration (54% vs. 31%, *p* = 0.02), and apoptosis (30% vs. 9%, *p* = 0.02) were more frequently found in patients with endoscopic activity. In contrast, eosinophilic infiltration was more prevalent in patients without endoscopic activity (1% vs. 12%, *p* = 0.003), as shown in Figure 2.

## 4. Discussion

This study represents the largest published series to date of patients with moderate to severe (grade 3–4) immune-mediated colitis (IMC). Our results provide detailed clinical, endoscopic, and histological characterization using standardized indices to ensure consistency in reporting. Moreover, this study includes the largest cohort to date with structured histological assessment and clearly demonstrates the discordance between clinical presentation and endoscopic severity, underscoring the need for objective tools to appropriately stratify disease severity.

Despite the growing use of immune checkpoint inhibitors, the literature on IMC remains limited, hindering the diagnostic and therapeutic approach to this condition. Although most cases of IMC are mild and managed symptomatically, severe presentations pose a significant clinical challenge.

Unlike other studies that include patients with all grades of IMC [21], our analysis focuses exclusively on the most severe forms (grades 3 and 4), which are associated with greater prognostic impact and a need for intensive intervention. These severe cases have been rarely studied, and to our knowledge, no previous series has specifically described their symptomatic profile. The homogeneity of the patient population is also a major strength of our study.

While diarrhea is the hallmark symptom, we observed a high frequency of rectal bleeding (nearly 40%) and significant abdominal pain (moderate or severe in 40% of patients). The median number of daily bowel movements was 8, clearly exceeding the severity thresholds established by classical indices such as Truelove and Witts [22]. The interquartile range further shows that 75% of patients had six or more bowel movements per day. Additionally, the presence of tachycardia (26%) and anemia (49%) supports the conclusion that these patients had clinically severe IMC requiring urgent specialist care.

Endoscopic findings have also been poorly characterized in prior studies, which typically involve small samples or focus solely on CTLA-4–based treatments [23,24]. These studies often include patients with a wide range of disease severity, diluting the analysis of the most severe cases [21,25,26].

In our study, we used the Mayo and UCEIS indices to standardize endoscopic evaluation, given the lack of validated scoring systems specifically for IMC. The lesions observed were mostly like those seen in ulcerative colitis, with loss of vascular pattern and mucosal lesions (aphthae and ulcers) predominating. Although the two endoscopic indices showed strong correlation with each other, their correlation with clinical symptoms was low (r < 0.4), indicating clinical–endoscopic dissociation. This discordance is also well known in other inflammatory bowel diseases, particularly Crohn’s disease, and even in ischemic colitis. In the case of IMC, there could be several explanations for the discrepancy between involvement and symptoms. Firstly, it could be because histological activity could be sufficient to cause symptoms, even in the absence of macroscopic lesions. Another possibility is that the lesions have not been located, but exist in other parts of the digestive tract, including the small intestine. In addition, diarrhoea is a symptom that can have multiple causes in a cancer patient, including other drugs, nutritional supplements, radiotherapy, etc.

Currently, the prognostic implications of endoscopic lesions in IMC remain unclear; however, some studies [13,25] suggest that the presence of such lesions may be associated with worse clinical outcomes, reinforcing the need for objective mucosal evaluation.

From a histological standpoint, there are no validated diagnostic criteria for IMC. While apoptosis has traditionally been considered a characteristic feature [11,23,27], it was present in only one-quarter of our samples. The wide range of histopathological findings in our study—including cryptitis, lymphocytic infiltration, crypt abscesses, and lamina propria expansion—highlights the heterogeneity of this condition.

Although immunological studies have proposed distinct pathogenic mechanisms in patients treated with anti-PD-1/PD-L1 versus anti-CTLA-4 agents [28,29,30,31], our results did not reveal meaningful endoscopic or histological differences between these groups, except for a higher frequency of lamina propria expansion in patients receiving CTLA-4 blockade. It is possible that immunologic differences are not reflected in histological changes detectable with conventional clinical techniques.

One of the most relevant findings of our study is that more than 75% of patients with no endoscopic activity still had histological evidence of inflammation. This emphasizes the importance of obtaining biopsy samples even in the absence of macroscopic lesions for confirming the diagnosis, given its difficulty and importance in establishing appropriate treatment. However, the prognostic implications of this histological inflammation without macroscopic manifestation are still unknown. Drawing a parallel with ulcerative colitis, it is possible that they increase the risk of relapse, but prospectively designed studies will be necessary to verify this.

It is also noteworthy that our cohort consisted of hospitalized patients requiring intravenous corticosteroids and/or immunosuppressive therapy. Despite this, between 15% and 25% of patients, depending on the scoring index used, had endoscopic scores consistent with remission. It is possible that in some of these cases, inflammation was localized to more proximal segments of the intestine that were not adequately examined, which could explain the persistent clinical symptoms.

One group of patients of particular interest is those who had previously been diagnosed with IBD. In these patients, it is unclear whether the symptoms are due to immune-mediated colitis or a reactivation of the underlying IBD. In our study, the number of patients with previous IBD is very small (8 patients) and does not allow us to obtain differential data with respect to the rest of the patients.

In our opinion, the results of this study reinforce the importance of performing an endoscopy with biopsies as early as possible to characterize the condition and refine the differential diagnosis as much as possible. This would allow targeted treatment to be started as soon as possible, with the aim of improving the patient’s clinical outcome.

Our study has several important strengths. First, the large sample size allows for precise characterization of the clinical and endoscopic features of severe IMC. The homogeneity of the cohort, restricted to grade 3–4 cases, reduces variability and enhances the robustness of our findings. Additionally, the multicenter design increases the generalizability of the results and minimizes selection bias.

Nonetheless, there are limitations. The retrospective design introduces potential biases in data collection. Some groups, such as patients receiving CTLA-4, are small, which limits the ability to find differences. The lack of standardized criteria for endoscopic and histological assessment adds heterogeneity to the interpretation of findings, although this was mitigated using structured reporting. Finally, data on the extent of endoscopic involvement were not available, and not all patients underwent endoscopy. The extent and distribution of endoscopic involvement could not be determined, which is an additional limitation. Endoscopic and histological tests have been read locally, which increases variability due to interobserver differences.

Furthermore, there is no validated index for symptoms, so we have used an adapted version of the partial May index that includes pain to systematize the results. However, this is an exploratory index that has not been validated and will require further investigation. In this study, clinical results are not available, which limits the information obtained. However, the main objective is to comprehensively characterize the most severe cases, enabling clinicians to identify them correctly and assisting in the systematic characterization of cases to improve patient management.

In conclusion, this study provides clinically relevant evidence on the most severe forms of IMC. It reveals notable heterogeneity in clinical, endoscopic, and histological expression, with limited correlation among these dimensions. These findings highlight the urgent need to develop specific clinical, endoscopic, and histological tools to support the comprehensive assessment of this emerging condition. The heterogeneity of IMC suggests that multiple factors are involved that may modulate the condition. In addition to endoscopic and histological characterization, other dimensions, such as the role of the microbiota or the patient’s immunological phenotype, should be considered in future prospective studies. Furthermore, genetic factors or factors linked to visceral adipose tissue should also be included in the comprehensive assessment of these patients.

## Figures and Tables

**Table 1:** Baseline characteristics of the cohort.

Variable	Overall (N = 196)
**Sex**	
Woman	79 (40.3%)
**Age**	
Mean (SD)	62.7 (11.4)
Range	20.0–89.0
**Cardiovascular disease**	
Yes	11 (5.6%)
**COPD**	
Yes	26 (13.3%)
**Diabetes mellitus**	
Yes	42 (21.4%)
**Hypertension**	
Yes	83 (42.3%)
**History of IBD**	
Yes	8 (4.1%)
**Smoking status**	
Active smoker	50 (25.5%)
Former smoker	80 (40.8%)
Never smoker	66 (33.7%)
**Obesity**	
Yes	15 (7.7%)
**Tumor location**	
Breast	5 (2.6%)
Colon	8 (4.1%)
Genitourinary	20 (10.2%)
Gynecologic	6 (3.1%)
Head and neck	7 (3.6%)
Liver	11 (5.6%)
Lung	66 (33.7%)
Melanoma	58 (29.6%)
Other	5 (2.6%)
Upper GI	10 (5.1%)
**Tumor stage**	
Metastatic	135 (68.9%)
**Mechanism of action**	
CTLA-4	42 (21.4%)
CTLA-4+PD1	4 (2.0%)
PD1	121 (61.7%)
PDL1	29 (14.8%)
**Concomitant therapies**	
Chemotherapy	66 (33.7%)
Targeted therapy	44 (22.4%)
**Immunotherapy cycles**	
Median (IQR)	4 (2–8)
**Time from immunotherapy start to symptoms**	
Median (IQR)	76.5 (28–197)
**Prior corticosteroid use**	
Systemic	81 (41.3%)
Topical	5 (2.6%)
COPD: Chronic obstructive pulmonary disease	

**Table 2:** Symptom characteristics at hospital admission.

Variable	N = 196
**Blood in stool**	
Yes	76 (39%)
**Abdominal pain**	
Absent	48 (24%)
Mild	69 (35%)
Moderate	75 (38%)
Severe	4 (2%)
**Bowel movements/24 h, median (IQR)**	8 (6, 10)
**General condition**	
Good	13 (6.6%)
Mildly impaired	50 (26%)
Moderately impaired	117 (60%)
Severely impaired	16 (8.2%)
**Tachycardia**	
Yes	48 (26%)
**Hemodynamic instability**	
Yes	16 (8.2%)
**Anemia**	
Yes	96 (49%)

**Table 3:** Endoscopic findings.

Variable	N = 139
**Loss of vascular pattern**	
Partial	38 (27%)
Total	73 (53%)
No	28 (20%)
**Mucosal bleeding**	
Significant spontaneous	4 (2.9%)
Mild spontaneous	20 (14%)
On contact	50 (36%)
None	65 (47%)
**Mucosal lesions**	
Deep ulcers	21 (15%)
Superficial ulcers	30 (22%)
Aphthae	45 (32%)
None	43 (31%)
**Mayo endoscopic score**	
3	51 (37%)
2	45 (32%)
1	22 (16%)
0	21 (15%)
**UCEIS classification**	
Severe	12 (8.6%)
Moderate	30 (22%)
Mild	61 (44%)
Remission	36 (26%)

**Table 4:** Histological findings.

Variable	N = 141
**Crypt abscesses**	
Yes	52 (37%)
**Cryptitis**	
Yes	71 (50%)
**Lymphocytic infiltration**	
Yes	68 (48%)
**Apoptosis**	
Yes	36 (26%)
**Lamina propria expansion**	
Yes	37 (26%)
**Collagen band thickening**	
Yes	5 (3.5%)
**Eosinophilic infiltration**	
Yes	5 (3.5%)
**Basal plasmacytosis**	
Yes	9 (6.4%)

## Data Availability

The data presented in this study are available on request to the corresponding author with prior authorisation of our Ethical Committee, which can be obtained at [https://www.iacs.es/investigacion/comite-de-etica-de-la-investigacion-de-aragon-ceica/ceica-evaluaciones-y-otras-presentaciones/], accessed on 14 April 2025.

## Abbreviations

The following abbreviations are used in this manuscript: CTCAE: Common Terminology Criteria for Adverse Events; GETECCU: Grupo Español de Trabajo en Enfermedad de Crohn y Colitis Ulcerosa; IBD: Inflammatory Bowel Disease; ICI Immune Checkpoint Inhibitors; IMC: Immunomediated Colitis; UC: Ulcerative colitis: UCEIS: Ulcerative Colitis Endoscopic Index of Severity
